# Costs associated with adverse events among acute patients

**DOI:** 10.1186/s12913-017-2605-5

**Published:** 2017-09-13

**Authors:** Jakob Kjellberg, Rasmus Trap Wolf, Marie Kruse, Susanne R. Rasmussen, Jesper Vestergaard, Kent J. Nielsen, Kurt Rasmussen

**Affiliations:** 1 KORA, the Danish Institute for Local and Regional Government Research, Copenhagen and Aarhus, Købmagergade 22, 1150 Copenhagen K, Denmark; 2 Department of Occupational Medicine, University Research Clinic, Herning Regional Hospital, Herning, Denmark

**Keywords:** Adverse events, Hospital costs [MeSH], Cost and cost analysis [MeSH]

## Abstract

**Background:**

The aim of this study was to analyse the additional treatment costs of acute patients admitted to a Danish hospital who suffered an adverse event (AE) during in-hospital treatment.

**Methods:**

A matched case-control design was utilised. Using a combination of trigger words and patient record reviews 91 patients exposed to AEs were identified. Controls were identified among patients admitted to the same department during the same 20-month period. The matching was based on age, gender, and main diagnosis. Cost data was extracted from the Danish National Cost Database for four different periods after beginning of the admission.

**Results:**

Patients exposed to an AE were associated with higher mean cost of EUR 9505 during their index admission (*p* = 0.014). For the period of 6 months from the beginning of the admission minus the admission itself they were associated with higher mean cost of EUR 4968 (*p* = 0.016). For the period from the 7th month until the end of the 12th month there was no statistically significant difference (*p* = 0.104). For the total period of 12 month, patients exposed to an AE were associated with statistically significant higher mean cost of EUR 13,930 (*p* = 0.001).

**Conclusions:**

AEs are associated with significant hospital costs. Our findings suggest that a follow-up period of 6 months is necessary when investigating the costs associated with AEs among acute patients. Further research of specific types of AEs and the costs of preventing these types of AEs would improve the understanding of the relationship between adverse events and costs.

## Background

Patient safety is a constant focus for health care workers and policymakers throughout the world. A study from 2001 found that adverse events (AEs) occur in 5.3% of all admittances to Danish hospitals. Forty percent of these AEs were judged by clinicians to have been preventable [1]. AEs cause physical harm, prolonged hospital stays and permanent injuries to patients. As a consequence, AEs are associated with increased healthcare costs, however the magnitude of these additional costs is not well known.

Rising healthcare costs increase the pressure on policymakers to prioritise in healthcare. Interventions to improve patient safety are often being refused due to the high implementation costs. Further insights into the actual costs of AEs might increase the willingness to invest in interventions, not only based on a healthcare perspective but also on an economic perspective.

Most studies on the costs associated with AEs have focused on particular categories of events, such as medical errors and diagnostic entities [2–13], e.g. sepsis. These studies which are primarily from the US and Australia find relatively different costs associated with AEs ranging from around EUR 650 to around EUR 35,000 per AE. The variation in costs can partly be explained by methodological differences, but they also emphasise that the costs of AEs vary between both type and context of the AE. The only four studies that have investigated the cost of an average AE across the different medical specialties found that AEs caused additional mean costs of around EUR 2800 to EUR 6100 [14–17].

In three of the four previous studies investigating the cost of an average AE patient record reviews were carried out to identify AEs [15–17]. Although this is a very time consuming task, the method ensures that actual AEs have occurred. In one of the studies, a database was scanned for a prefix indicating that a diagnosis had occurred during the admission. Those diagnoses were then assessed as being the result of AEs [14]. This approach makes it easier to identify many AEs, but the precision of the identification is likely to be lower than when AEs are identified with patient record reviews by clinicians. The studies that identify the AEs utilising patient record review also determined the additional healthcare costs based on the reviewers estimation of extra bed days and procedures combined with standard rates [15–17]. This approach gives an approximation of the costs, but due to the standard rates, which are likely to differ when dealing with patients exposed to AEs, it is probably not very precise. The aforementioned study utilised a patient-level cost database, which enabled the researchers to use the actual patient costs and determine the costs associated with the AEs based on a case-control design [14].

The aim of this study was to calculate the additional treatment costs of emergency department (ED) patients who suffered an AE during in-hospital treatment. This was done using trigger words for identification of potential AEs and patient record reviews for verification of AEs, combined with a patient-level cost database to determine costs and to identify a control population. This approach combines a high precision in identification of AEs with a precise estimate of hospital costs, and to the best of our knowledge makes this study the first ever to estimate the cost of adverse events by combining verified adverse events and actual patient costs. Furthermore, this study analyses both the costs during the admission where the AE occurred and the patients total hospital costs within the following 12 months. This is done in order to investigate both whether AEs are associated with additional costs during the admission as well as after the initial discharge.

## Methods

### Setting

The study was performed at a Danish regional hospital among acute patients requiring services from the following specialties: emergency medicine, general surgery, orthopaedic surgery and internal medicine (except cardiology). The patients’ initial contact with the hospital was through the ED, which comprised an emergency admission and a bed ward. The patients were diagnosed and treated at the ED with support from physicians from other hospital departments. The ED had a catchment area of approximately 300,000 people and served 21,000 patients a year. The mean number of admissions to the ED was approximately 60 patients per day, varying between about 30 to 120 patients from day to day, and about 50% of patients were admitted to the bed ward. Around 70% were discharged from the ED within 24 h. The remaining 30%, which were the most severe cases, were transferred to the two surgical departments or the department of internal medicine for further treatment.

### Identification of cases

An initial sorting of patient records was carried out using an electronic trigger tool inspired by The Global Trigger Tool [18]. The trigger tool automatically scans records for predefined trigger words and returns the number of identified triggers in each record. The trigger tool was developed at the department where the study was performed and have gone through a validation procedure (face, content and construct validity) ending up with 42 trigger words (trigger words listed in Table 1). To reduce the number of patient records that had to be read in full text only patient records with at least seven trigger words were selected for full record review.

**Table 1:** Distribution of trigger words by SAC score

Trigger word^a^	SAC score			Total (%)
	Intermediate	Severe	Catastrophic	
Accidently	1	0	0	1 (1)
Angioedema	0	1	0	1 (1)
Discovered	1	0	0	1 (1)
Error	6	2	0	8 (9)
Does not exist	2	0	0	2 (2)
Glucose	1	0	0	1 (1)
Has not been	1	0	0	1 (1)
Has not received	1	0	0	1 (1)
However not received	1	0	0	1 (1)
It	1	0	0	1 (1)
Intensive	0	1	0	1 (1)
Missing	4	2	0	6 (7)
Narcanti	1	0	0	1 (1)
Not agree	0	1	0	1 (1)
Not available	4	0	2	6 (7)
Not existing	3	0	0	3 (3)
Not possible	1	0	0	1 (1)
Not received	5	2	1	8 (9)
Not registered	1	0	0	1 (1)
Problems	1	0	0	1 (1)
Prolonged	0	1	0	1 (1)
Readmission	1	1	0	2 (2)
Readmitted	1	0	0	1 (1)
Shows	4	2	0	6 (7)
Tried	2	0	0	2 (2)
Unable to get	2	1	0	3 (3)
Unfortunately	17	5	0	22 (24)
Was readmitted	3	0	0	3 (3)
Wrong	2	2	0	4 (4)
Total (%)	67 (74)	21 (23)	3 (3)	91 (100)

^a^Trigger words are translated from Danish unless international medical terms were used.

Patient records from the period of 30st April 2010 to 31st December 2011 were scanned with the trigger tool. The identified patient records with at least seven trigger words were read by a senior medical doctor (the last author of the paper) to evaluate if there was an AE. Furthermore, the seriousness of the AE was assessed using the Safety Assessment Code (SAC) [19]. This is an internationally used 4 point scoring system ranging from minor to catastrophic (fatal). In the present study, only AEs rated as intermediate, severe or catastrophic were included. Complications, defined as (ordinarily) rare unintended sequelae despite correctly performed procedure (as established by the national clinical guidelines), were excluded from the material. It was not possible to distinguish between preventable and non-preventable AE cases in this setting.

### Matching

A matched sample of cases and controls were used to determine the differences in associated hospital costs. Cases were the identified patients with an AE.

We selected a control group, comprising patients that had contact with the same regional hospital during the same period but who were not exposed to an identified AE. Cases were matched with controls using exact matching on three parameters: age (10 year intervals), gender and main diagnosis. The number of controls per case was not fixed, as explained below. .

In order to maximise the number of matches, matching on age was based on 10-years intervals. To check for discrepancies with the distributions within the intervals, the mean age of cases and controls were compared. This comparison did not raise any concerns.

The main diagnosis is given at discharge by the physician in charge and is the diagnosis that best describes treatment conducted at the hospital. The diagnoses are coded according to the Danish version of the International Classification of Diseases, 10th revision (ICD-10).

### Costs

Cost data used in this study were extracted from the Danish National Cost Database, which contains data regarding patient-level hospital costs. The National Cost Database provides the total cost for every patient discharged from any public hospital in Denmark based on the patient’s actual utilisation of hospital services. All cost data were converted to Euros (EUR) using the 2011 exchange rate (EUR 1 = DKK 7.45 [20]).

For each case and control, cost data were extracted from the first day of the admission of interest (index admission) and the following 12 months. This not only allowed the analysis of cost difference for the primary admission but also allowed us to analyse the differences in cost of hospital services used in the following months after the discharge.

As no information on comorbidity was available, cost data from the 12 months prior to the primary admission were also extracted for all patients. These cost data were used as a proxy for comorbidity as we hypothesised that relevant comorbidity will be expressed by higher hospital costs in this period. The hospital costs in the 12 months period before the primary admission were compared between the case and the control groups to check if the two populations were comparable.

### Statistics

All analysis was undertaken using Stata version 13 [21].

The number of controls varied for each case. Hence, the controls were weighted and presented in the results as the inverse of the number of controls for the case they match (1/n_number of controls for case_).

Means of cost data for cases and controls were calculated with 95% confidence intervals (CIs). We used non-parametric bootstrapping with 1000 replications to calculate CIs of the cost estimates, because the cost data followed a non-normal distribution and were skewed to the right [22]. Non-parametric bootstrapping is the preferred method to estimate 95% CIs around cost-estimates because it uses the distribution of the data rather than assume a normal distribution [23].For comparison of mean costs between cases and controls, bootstrap t-test with 1000 replications was used, as using a bootstrapped t-distribution does not require assumptions of normal distribution [23].

## Results

**Fig. 1:**
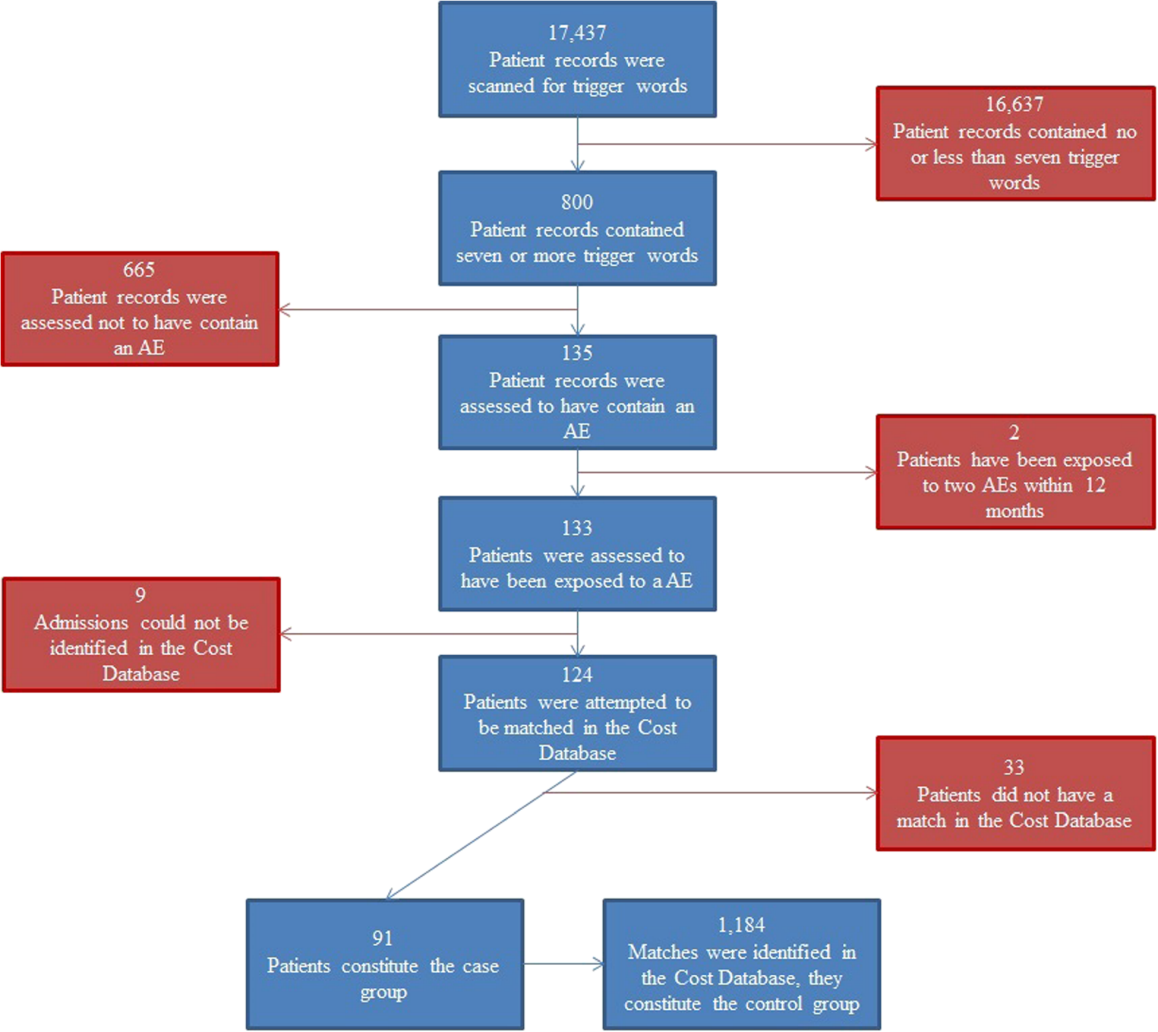
Study flow

In total 17,437 records from the period of 1th May 2010 to 31st May 2011were scanned with the electronic trigger tool. Eight hundred records with up to 21 trigger words per record were identified and read in full text, and 135 cases with AEs were identified. Of these, two individuals had two admissions with AEs within 12 months. The two most recent cases of AEs were excluded due to the study’s 12 months focus. Nine cases were excluded due to lack of information in the Danish National Cost Database. Thirty three cases were excluded as it was not possible to find any matching controls, the major limiting factor in these cases were the main diagnoses. In total, 91 cases were included in this study. For each included case, we were able to identify 1–93 matching controls. In total, 1184 controls were included in the analysis. Figure 1 illustrates the study flow.

In the patient records of the 91 cases, up to 21 trigger words were found. On average, each of these patient records contained ten trigger words. Table 1 shows which trigger words the 91 AEs in the cases have been identified by and the severity of the AEs assessed with SAC. The trigger word “unfortunately” was the most frequent as 24% of the AEs were identified by this word. 74% of the total AEs were assessed to be intermediate, 23% severe and 3% catastrophic.

The 91 cases and 1184 control patients had 68 different main diagnoses, representing 12 of the 21 ICD-10 chapters. Diseases of the digestive system and the respiratory system were the most common amounting to 24% (22 cases) and 19% (18 cases) respectively.

We compared the baseline characteristics of the cases and controls (see Table 2). Due to matching, both groups consisted of 52 women (57%) and have a mean age of 66 years. As the matching was based on 10-years intervals, there could have been a larger difference in the mean age between the two groups. However, there was no significant (*p* = 0.965) difference which strengthens the validity of the matching. To check for selection bias, hospital cost data from the 12 months before the primary admission were compared for cases and controls. Cases had an insignificant (*p* = 0.658) EUR 618 higher mean cost during this period. Even though no final conclusions can be made, the insignificant difference in costs further strengthens the assumption of the two groups being comparable at baseline.

**Table 2:** Baseline characteristics of cases and controls

	Cases	Controls (weighted)	*p*-value
Patients (n)	91	91 (1184 non-weighted)	
Female (n)	52	52	
Age (mean)	66.3	66.4	0.965
Cost: 12 months before primary admission, mean EUR [95% CI]	5383 [2998–7768]	4765 [3561–5969]	0.658

**Fig. 2:**
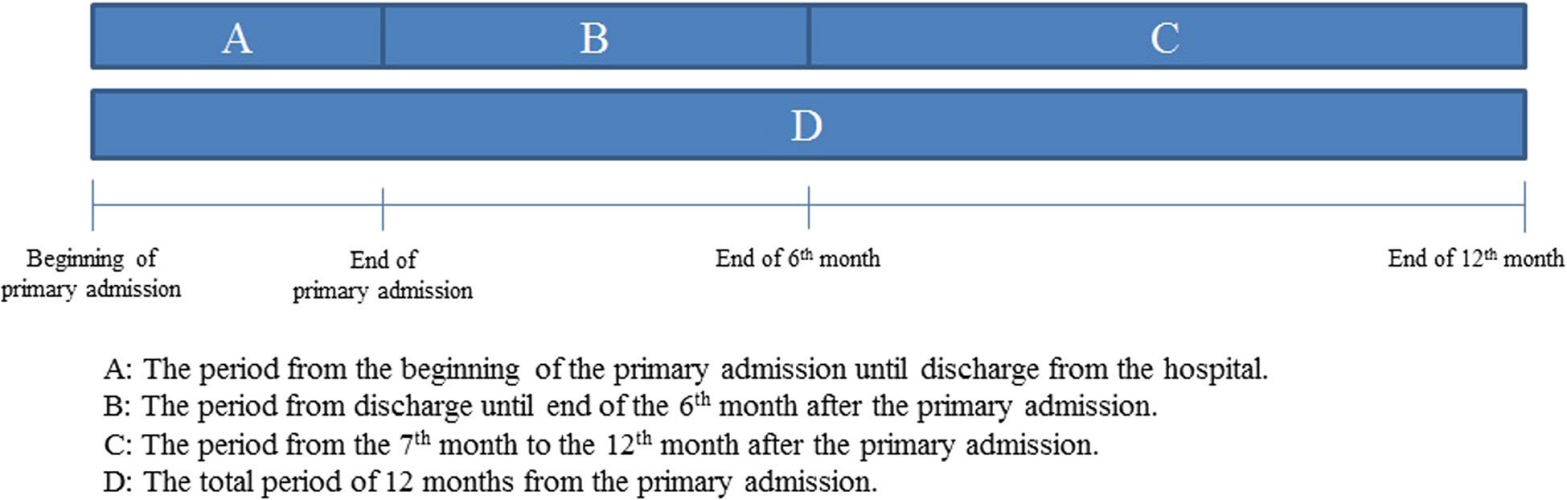
The different costs periods

The difference in hospital costs between cases and controls were analysed for four different periods (see Fig. 2): the primary admission (A), 6 months from the primary admission minus the primary admission itself (B), the 7th month--the 12th month after the primary admission (C) and the total period from the primary admission and 12 month onwards (D).

We compared the means for cases and controls during these periods (see Table 3). We found the mean costs of the primary admission (period A) to be EUR 18,826 for cases and EUR 9321 for controls. Thus, the mean costs of the primary admission were 102% higher for patients with an AE compared to patients who did not experience an AE. The difference was statistically significant (*p* = 0.014).

**Table 3:** Cost of cases and controls

	Cases	Controls (weighted)	*p-value*
Patients	91	91 (1184 non-weighted)	
A. Cost: Primary admission, mean EUR [95% CI]	18,826 [12,417–25,235]	9321 [6867–11,775]	0.014
B. Cost: 6 months from primary admission minus cost of primary admission, mean EUR [95% CI]	8607 [4977–12,237]	3639 [2791–4488]	0.016
C. Cost: 7th to 12th month after the beginning of the primary admission, mean EUR [95% CI]	623 [156–1090]	1166 [753–1047]	0.104
D. Cost: Total cost from beginning of primary admission and 12 month onwards, mean EUR [95% CI]	28,056 [20,997–35,115]	14,126 [11,295–16,957]	0.001

For the rest of the 6 months period beginning at the primary admission (period B), we also found the mean costs for cases to be statistically significantly higher (*p* = 0.016) than for controls. The mean cost of cases is EUR 8607 and EUR 3639 for controls. The mean cost is EUR 4968 higher for patients with an AE compared to patients who did not experience an AE.

In the 7th - the 12th month after the primary admission (period C), we found a non-statistically significant (*p* = 0.104) EUR 543 lower mean cost for cases.

For the total period, from the beginning of the primary admission and 12 months onwards (period D), we found the mean cost of cases to be EUR 28,056 and EUR 14,126 for controls. The statistical significant (*p* = 0.001) higher mean costs of EUR 13,930 rendered the mean cost for the 12 month period 99% higher for patients with an AE compared to patients, who did not experience an AE.

## Discussion

AEs of moderate or higher severity were associated with higher hospital costs both during the admission when the AE occurred and during the rest of the period from the admission and 6 months onwards. For the period from the beginning of the 7th month through the end of the 12th month following the initial admission, we found that not being exposed to an AE was insignificantly associated with higher costs. These findings suggest that a follow-up period of 6 months is sufficient when investigating the costs associated with AEs.

We found that the mean cost of the index admission was EUR 9505 higher for patients exposed to an AE compared to the mean cost of patients not exposed to an AE. For the full 12 months period, we found that the mean cost is EUR 13,930 higher for patients exposed to an AE. We therefore found that for the full 12 months period, the mean cost of a patient exposed to an AE was 99% higher than the mean cost of a patient not exposed to an AE. Of this amount, 36% was associated with the period after discharge. This emphasises the need for a follow-up period after discharge to determine the actual hospital costs of an AE.

Our findings are considerably higher than those of the previous four studies mentioned in the introduction [14–17], according to which the extra mean cost associated with an AE was EUR 2800–6100. Out of the four studies, only one [15] took readmissions into account when calculating the extra costs. According to our findings, this implies that the three other studies are likely to have underestimated the actual additional costs. Other probable explanations for the higher costs associated with AEs are inflation and general increases in medical treatment costs, since the other studies were conducted between 2001 and 2009.

Out of the 17,427 patient records that were part of this study, we identified 800 patient records with at least seven words of which 135 contained an AE of moderate or higher severity. The 800 patient records represent only 0.5% of the total number of records scanned with the electronic trigger tool, and it is likely that a considerable number of the remaining 99.5% will contain AE’s. The true number of AE’s is unknown. Previous studies have shown an association between the impact of work related stressors and the involvement in adverse events for both doctors and nurse [24, 25]. These specific characteristics of the work environment at ED’s combined with the acute medical activities might result in EDs being specifically prone to AEs. Thus, some of the 1184 control patients will probably have been exposed to AEs. This will led to our findings underestimating the additional costs of AEs.

Due to the limited number of cases, it was not meaningful to analyse the costs by specific ICD-10 groups. This is a limitation when considering generalisation of the results. The only knowledge we have of the AEs characteristics are based on the ICD-10 distribution and the distribution in the SAC assessment. By comparing these to the ICD-10 distribution of all admissions to Danish hospitals [26] and the distribution of SAC of the AEs reported to the mandatory system [27], we found that the distributions are roughly comparable, except for the orthopedic area.

There was no information of severity at baseline admission available. This is a limitation of the study as severity at admission could be a potential confounder both influencing the cost and risk of AE.

In order to estimate the total annual Danish national cost associated with AEs at hospitals, it is necessary to estimate the total amount of AEs. It is generally acknowledged that far from all AEs are reported to the national mandatory system. A Danish study compared voluntary AE-reporting to the mandatory reporting system with daily end-of-shift registration, and found that only 5% of the AEs were reported to the mandatory system, with no differences in the severity of the AE’s reported [24]. By combining our finding that an AE costs EUR 13,930 with the assumption that 5% of all AEs are reported to the mandatory system, the estimated total cost of AEs per annum for the Danish Hospitals is about EUR 3.1 billion based on the number of AEs reported to the mandatory system in 2013 [27] Even though this is a very rough estimate as it is based on assumptions that costs of AEs are the same in acute and non-acute patients and assumptions of the actual number and characteristics of AEs annually taking place in Denmark, this suggests that there is a strong economic perspective in introducing interventions to prevent AEs despite the immediate costs of these.

The findings of substantial cost associated with AEs underlines the need for future studies that identify preventable AEs and the cost of these. The present study suggest that the combination of a trigger tool and patient record reviews can be a useful approach for the identification of actual AEs in future studies. The results of the study furthermore shows that future studies should not only focus on the cost during the admission with the AE but at least on a 6 month period from the beginning of the admission to make sure all costs due to the AE are taken into account.

Further research into the healthcare costs of specific types of AEs and into the costs of preventing these exact types of AEs would open an opportunity of conducting economic evaluations and hence present better insights into the possibilities of cost saving and quality improving interventions.

## Conclusion

Patients exposed to an AE had a statistically significant higher mean cost of EUR 13,930 for the total follow-up period of 12 month compared to patients not exposed to an AE.

## Abbreviations

AE: Adverse event
CIs: Confidence intervals
ED: Emergency department
EUR: Euro
ICD-10: The international classification of diseases, 10th revision
SAC: Safety assessment code
